# Management of encounters related to subfertility and infertility in Australian general practice: a focus on Aboriginal and Torres Strait Islander females

**DOI:** 10.1186/s12905-023-02559-x

**Published:** 2023-08-04

**Authors:** E Gilbert, A. Rumbold, S. Campbell, J. A. Boyle, L. Grzeskowiak

**Affiliations:** 1 Faculty of Health, Charles Darwin University, Darwin, NT Australia; 2 SAHMRI Women and Kids, South Australian Health and Medical Research Institute, Adelaide, SA, Australia; 3 Eastern Health Clinical School, Monash University, Melbourne, VIC Australia; 4 College of Medicine and Public Health, Flinders University, Adelaide, SA, Australia

**Keywords:** General practice, Aboriginal and Torres Strait Islander, Indigenous, Infertility, Fertility, Reproductive health

## Abstract

**Objective:**

To investigate the management of subfertility and infertility among Aboriginal and Torres Strait Islander females attending Australian general practice.

**Methods:**

Cross-sectional study of 1,258,581 women (18–49 years) attending general practice between January 2011 and June 2019, utilising data from NPS MedicineWise MedicineInsight, a national general practice database in Australia.

**Results:**

The prevalence of subfertility/infertility encounters was lower for Aboriginal and Torres Strait Islander females (12.37 per 1,000) than for non-Indigenous females (16.62 per 1,000). Aboriginal and Torres Strait Islander females with a subfertility/infertility encounter were younger and more likely to live outside Major cities and in areas of socioeconomic disadvantage than non-Indigenous females. Rates of prescribed infertility medications were not different between groups, however Aboriginal and Torres Strait Islander females were more likely to receive a pelvic ultrasound (24.30% vs. 19.90%); tests for luteinizing hormone (31.89% vs. 25.65%); testosterone (14.93% vs. 9.96%) and; glycated haemoglobin (HbA1c) (6.32% vs. 3.41%),but less likely to receive an anti-müllerian hormone test (2.78% vs. 7.04%).

**Conclusions:**

Lower encounter rates for infertility/subfertility among Aboriginal and Torres Strait Islander peoples may indicate access issues, preferred use of Aboriginal community-controlled health centres or younger average age at first birth and thus less age-related infertility.

**Implications for public health:**

Future efforts should focus on maximising the inclusiveness of infertility surveillance. There is also a need for further research into the experiences of and preferences for infertility care and associated barriers among Aboriginal and Torres Strait Islander people.

## Introduction

Infertility is defined as the inability to achieve a pregnancy after 12 months or more of regular unprotected sexual intercourse in a woman aged < 35 years or 6 months in a woman aged > 35 years [1]. Subfertility generally describes any form of reduced fertility with a delay in conceiving [2].

In Australia, infertility is estimated to affect 16% of reproductive-aged couples [3]. However, little is known about the prevalence of infertility within certain subgroups, including among Aboriginal and Torres Strait Islander peoples. One previous study reported that the rate of infertility in Aboriginal women in the Northern Territory could be as high as 26% [4], but this has not been confirmed in other jurisdictions nor is there comprehensive evidence about patterns of health care utilisation for infertility among Aboriginal and Torres Strait Islander women or men.

General practitioners (GPs) play a central role in the Australian healthcare system. They are often the first point of contact for people with a health issue, including for infertility-related concerns [5]. Currently, there are no national guidelines to support GPs in managing infertility. Thus, management is based on best available evidence and clinical expertise, typically involving an initial assessment compromising a review of the medical history and physical examination of the woman and her partner (if appropriate) to help direct further investigation and management [6]. This should include an evaluation of ovulation, ovarian reserve, and pelvic anatomy, semen volume and sperm quality. GPs can initiate these investigations by requesting pathology or radiology diagnostic tests (e.g., luteal progesterone, pelvic ultrasound). Management strategies may include psychosocial support, patient education, ovulation induction medications, and the treatment of comorbid conditions [6, 7]. Following initial assessment and preliminary investigations, GPs may refer patients to a fertility clinic or relevant specialist (e.g., gynaecologist or urologist) in either the private or public hospital systems for further management or in some instances, may continue management themselves (e.g., prescribing metformin).

Despite the central role GPs play in the management of infertility, there has been limited prior evaluation of infertility management in this setting in Australia. One previous study utilising national general practice data from the Bettering the Evaluation and Care of Health (BEACH) programme [8] reported the rate of infertility consultations as 28.3 per 1,000 women aged 18–49 years and 10.2 per 1,000 men aged 18–49 years [9]. When stratified by Indigenous status, Aboriginal and Torres Strait Islander patients had lower rates of infertility encounters than non-Indigenous patients (3.9 per 1,000 consultations vs. 5.9 per 1,000 consultations). However, the reliability of these findings was limited by the underrepresentation of Aboriginal and/or Torres Strait Islander patients in the BEACH dataset compared to national data (1.5% vs. 3.3%). Given Aboriginal and Torres Strait Islander peoples are disproportionally affected by a range of risk factors for infertility (e.g., polycystic ovary syndrome, sexually transmissible infections) compared to all Australians [10], accurate and reliable data for this population group is critical.

This study utilises national data from the NPS MedicineWise MedicineInsight dataset [9], which is broadly representative of the Australian population including Aboriginal and Torres Strait Islander peoples. The aim was to investigate the management of subfertility and infertility among Aboriginal and Torres Strait Islander females and non-Indigenous females attending general practices, including the frequency of presentations, the health and sociodemographic profile of those attending, the investigations undertaken, and medications prescribed to manage these encounters.

## Materials and methods

### Ethics

Access to the data from this study was approved by the MedicineInsight Data Governance Committee (project 2019-003). The Human Research Ethics Committee of the University of Adelaide exempted this study from ethical review due to the use of non-identifiable data.

### Study design, setting and, data source

This was a cross-sectional study using data from the NPS MedicineWise MedicineInsight dataset. The study period spanned 1 January 2011 to 31 June 2019. MedicineInsight is a large-scale, national general practice dataset established by NPS MedicineWise with core funding from the Australian Government Department of Health. The MedicineInsight dataset has been described in detail elsewhere [11]. In summary, MedicineInsight uses third-party extraction tools (GRHANITETM and Precedence Health Care’s cdmNetTM) which extract, de-identify and securely transmit patient data from participating practices’ clinical information systems (CISs), such as Best Practice and Medical Director, to a secure data repository. The extraction tool collects incremental data regularly, allowing the development of a longitudinal database in which individuals within each practice can be tracked over time. The MedicineInsight dataset collects data on individual demographics, practice encounters (not including progress notes), diagnoses, prescribed medication, pathology tests and, referrals. Insights are enriched through selected free text data.

On July 1, 2019, the dataset included records for approximately 3.5 million regular patients (approximately 15% of the Australian population) from 715 general practices and more than 5000 GPs across Australia. The characteristics of MedicineInsight patients have been demonstrated to be broadly representative of the Australian population, including in relation to Aboriginal and Torres Strait Islander background (3.0% vs. 3.3%) [12].

### Study population

For this study, we restricted our analysis to females^1^ of reproductive age (18–49 years inclusive). To improve data quality, we restricted patients to those with two or more encounters (for any reason) during the study period, as they were more likely to reflect people who had a closer relationship with that particular practice and improve the quality of recorded data pertinent to the project (e.g., Indigenous status, smoking status, co-morbidities etc.).

**Fig. 1:**
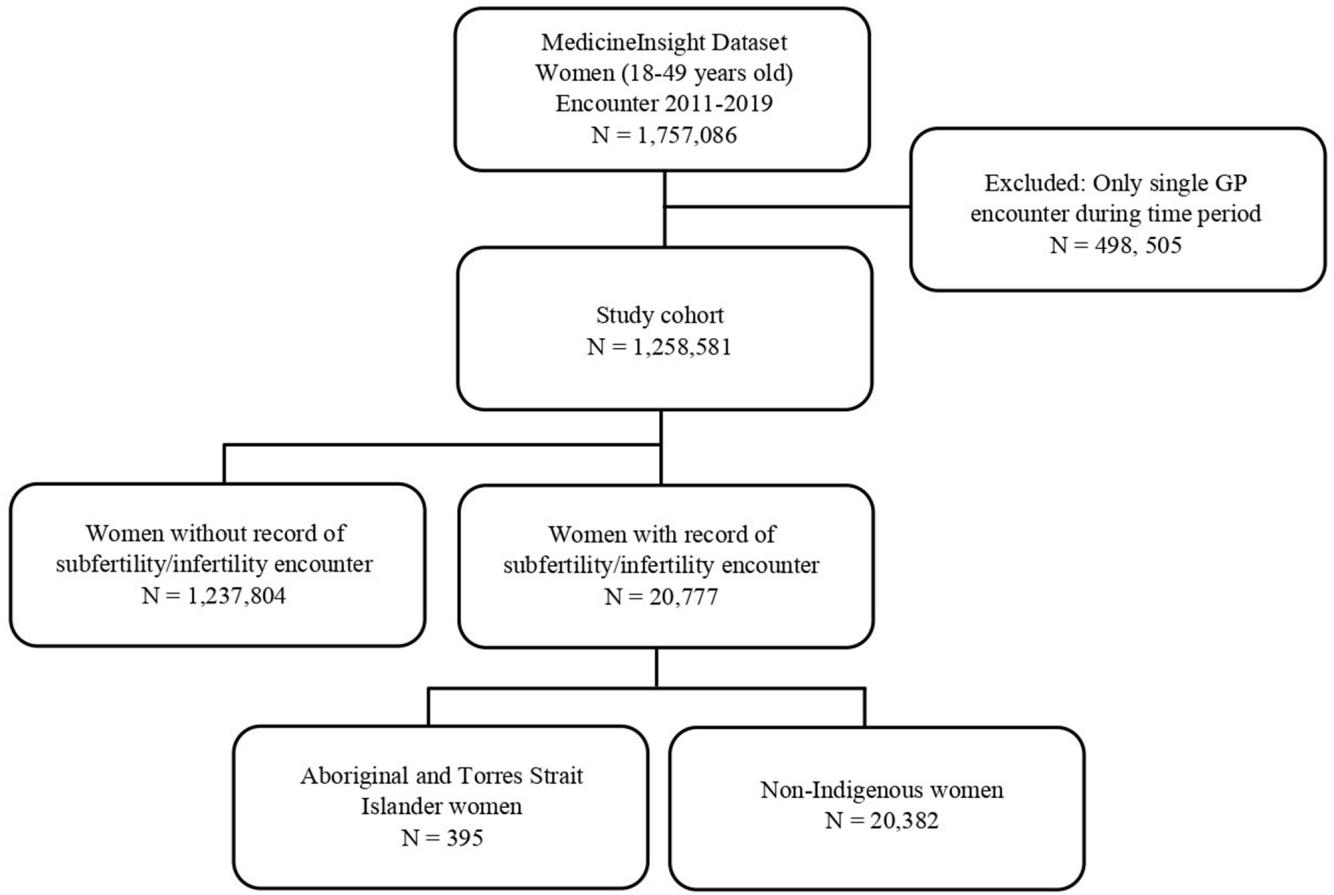
Flow chart for selection of the main study population and subfertility/infertility population

Females with concerns of subfertility/infertility were identified as those who had codes (Docle, Pyefinch or ICPC-2 PLUS medical condition coding) or free text in the field ‘reason for encounter’ that indicated subfertility/infertility during the study period. The search terms included: ‘infertility’, ‘subfertility’, ‘impaired fertility’, ‘fertility problem/issue’, ‘ovarian dysfunction’, ‘unable to get pregnant’, ‘in vitro ‘fertilisation’ and ‘artificial insemination’, as well as synonyms and possible misspellings of these words. The data extraction algorithms used in this study are available from the authors by request. Patients who met the above subfertility/infertility population criteria were further stratified by Aboriginal and Torres Strait Islander status. A study flow diagram is provided in Fig. 1.

Patient characteristics included age (based on year of birth), remoteness, socio-economic indexes for areas (SEIFA), state/territory, Aboriginal and Torres Strait Islander status, Commonwealth concession card status and smoking status. Females for whom Aboriginal and Torres Strait Islander status was recorded as unknown or missing were re-categorised as non-Indigenous. Remoteness, socio-economic indexes for areas (SEIFA) and state/territory were based on patients’ residential postcodes. Remoteness was determined in accordance with the Australian Bureau of Statistics’ (ABS) Australian Statistical Geography Standard (ASGS) remoteness areas, with 1 being a major city and 5 a very remote area. Due to small population sizes, data for ‘Inner Regional’ and ‘Outer Regional’ as well as ‘Remote’ and ‘Very Remote’ were combined for reporting purposed. SEIFA was determined according to the ABS Index of Relative Socio-Economic Advantage and Disadvantage (IRSAD) codes. We identified those who potentially travelled significant distances to attend clinic appointments by comparing the remoteness indicators according to each woman’s residence compared with the GP practice. Additional characteristics of the cohort included the presence of relevant comorbidities including polycystic ovary syndrome, diabetes, hypercholesterolaemia, hyperlipidaemia and hypertension, as well mental health conditions such as depression and anxiety. Patients were defined as having any of these clinical conditions based on ‘conditional flags’ provided by NPS MedicineWise MedicineInsight – ever recorded at time from the patients earliest record up to the download date. Practice characteristics included remoteness of practice, which was determined in the same way as patient remoteness, and state/territory.

### Study outcomes

We calculated the number and prevalence of all individuals with a clinical encounter related to subfertility/infertility. These were stratified by Aboriginal and Torres Strait Islander status, a range of demographic and clinical characteristics (e.g., age, socioeconomic status, rurality).

The relevant management actions provided at subfertility/infertility encounters included risk factor monitoring for blood pressure, body mass index and blood sugar levels, prescription of selected medications, selected pathology tests and imaging ordered. In the absence of international consensus guidelines or national clinical practice guidelines relevant to infertility treatment, infertility treatment in Australia is based on the best available evidence and clinical expertise [16], combined with the National Health and Medical Research Council’s (NHMRC) Ethical Guidelines on the use of Assisted Reproductive Technology in clinical practice and research [17]. Therefore, the management actions selected for presentation in this paper reflect those routinely performed for investigation of subfertility and infertility, as well as investigations commonly undertaken to promote preconception health care. This is based on the clinical expertise of the authors of this article.

### Statistical analysis

Descriptive statistics (counts, percentages and associated 95% confidence (CIs)) were used to describe the study population. Chi-square tests were used to compare categorical variables of the Aboriginal and Torres Strait Islander and non-Indigenous groups. To measure the relative difference in subfertility/infertility encounter rates, rate ratios were calculated by dividing the Aboriginal and Torres Strait Islander rate by the non-Indigenous rate for each stratum. All analyses were based on two-sided P-values, with p < 0.05 considered statistically significant. To preserve the privacy of individuals, results reported for 1–4 patients are reported as < 5. The statistical analysis was performed using STATA SE 16 (Stata, College Station, Texas) and the MedCalc program (MedCalc Software v20, Ostend, Belgium).

## Results

### Characteristics of the main study population

From January 2011 to June 2019, there were 1,757,086 females of reproductive age (18–49 years) eligible for inclusion. Of these, 498,505 had only one encounter during the time period and were therefore excluded, leaving a study cohort of 1,258,581 females, across 440 practices nationally (Fig. 1). There were 31,944 individuals who identified as Aboriginal and/or Torres Strait Islander, representing 2.5% of all patients.

### Characteristics of females with a subfertility/infertility encounter in the study period

Table 1 shows the sociodemographic characteristics of females with a subfertility/infertility encounter, by Aboriginal and Torres Strait Islander status. The total number of females with a record of a subfertility/infertility encounter over the study period was 20,777 (1.65%). Of these females, 395 identified as Aboriginal and/or Torres Strait Islander (1.90%). Aboriginal and Torres Strait Islander females were younger than their non-Indigenous counterparts. Aboriginal and Torres Strait Islander females were also most likely to reside in regional areas (60.50%) and in areas of greater socio-economic disadvantage.

### Subfertility/infertility encounter rates

Table 2 presents the prevalence of subfertility/infertility encounters stratified according to individual demographics and Aboriginal and Torres Strait Islander status. Overall, the highest prevalence was seen in females aged 30–34 years (30.64 per 1,000 females) and those living in major cities (17.35 per 1,000 females). Additionally, an increasing trend in prevalence with increasing SES status was observed, with the highest prevalence found among females living in the most advantaged socioeconomic quintile.

The prevalence of Aboriginal and Torres Strait Islander females with a subfertility/infertility encounter was lower (12.37 per 1,000 females) than for non-Indigenous females (16.62 per 1,000 females), reflecting a rate ratio of 0.74 (Table 2). When stratified by age, subfertility/infertility encounters were highest for Aboriginal and Torres Strait Islander females aged 25–29 years (20.47 per 1,000), and for non-Indigenous females aged 30–34 years (30.91 per 1,000). The rate among Aboriginal and Torres Strait Islander females aged 18–24 years was more than double the rate in non-Indigenous females of the same age group (RR: 2.16). For all other sociodemographic characteristics, the rate ratio was either one or below one, indicating Aboriginal and Torres Strait Islander females in that strata were less likely to present with subfertility/infertility concerns than non-Indigenous females in the same strata.

### Management of subfertility/infertility encounters

Table 3 presents the management actions provided to females at subfertility/infertility encounters. Aboriginal and Torres Strait females, compared to non-Indigenous females, were significantly more likely to have a record of a BMI (38.98% vs. 25.39%); receive a pelvic ultrasound (24.30 vs. 19.90); and tests for LH (31.89% vs. 25.65%), testosterone (14.93% vs. 9.96%) and HbA1c (6.32% vs. 3.41%); but significantly less likely to receive a MMR test (11.39% vs. 15.48%) or AMH test (2.78% vs. 7.04%). There were no differences in the prescribing of metformin, ovulation induction agents, or any other management actions between the two groups.

**Table 1:** Demographic and clinical characteristics of females with an encounter for subfertility/infertility, stratified by Aboriginal and Torres Strait Islander status

**Characteristics** ^**1**^	Aboriginal and Torres Strait Islander **(N = 395)**		**Non-Indigenous ** **(N = 20,382)**		
	**Number**	%	**Number**	%	P-value
***Patient***					
**Age group (years) at first subfertility/infertility encounter**					
18–24	105	26.58	1,310	6.43	< 0.001
25–29	115	29.11	3,862	18.95	
30–34	88	22.27	6,278	30.80	
35–39	49	12.40	5,354	26.27	
40–44	29	7.34	2,707	13.28	
45–49	9	2.27	871	4.27	
**Rurality** ^**2**^					
Major city	145	36.70	14,521	71.24	< .0001
Inner/Outer Regional	239	60.50	5,447	26.72	
Remote/Very Remote	10	2.53	306	1.50	
Missing	< 5	-	108	0.53	
**Relative socio-economic advantage and disadvantage (SEIFA quintiles)**					
First quintile (most disadvantaged)	109	27.59	2,431	11.93	< 0.001
Second quintile	131	33.16	3,288	16.13	
Third quintile	86	21.77	4,396	21.57	
Forth quintile	47	11.89	4,646	22.79	
Fifth quintile (most advantaged)	20	5.06	5,471	26.84	
**Commonwealth concession card**					
Yes	203	51.39	2,613	12.82	< 0.001
No	156	39.49	14,262	69.97	
Not recorded	36	9.11	3,507	17.21	
**Smoking status**					
Never smoked	113	28.60	9,224	45.26	< 0.001
Ex-smoker	82	20.76	4,617	22.65	
Smoker	181	45.82	4,739	23.25	
Not recorded	19	4.81	1,802	8.84	
**Comorbid conditions**					
Asthma	105	26.58	2,954	14.49	< 0.001
Anxiety	147	37.21	4,412	21.65	< 0.001
Depression	160	40.50	4,707	23.09	< 0.001
Hypertension	27	6.83	965	4.73	0.052
Hyperlipidaemia	9	2.37	340	1.67	0.350
Hypercholesterolaemia	11	2.78	541	2.65	0.873
Diabetes	19	4.81	353	1.73	< 0.001
Polycystic ovary syndrome	81	20.50	2,661	13.06	< 0.001
***Practice characteristics***					
**Practice location** ^**3**^					
Major city	137	34.68	14885	73.03	< 0.001
Inner/Outer Regional	249	63.03	5228	25.65	
Remote/Very Remote	9	2.37	269	1.32	
**State/Territory**					
Australian Capital Territory	< 5	-	716	3.51	< 0.001
New South Wales	227	57.46	8264	40.55	
Northern Territory	5	1.26	226	1.11	
Queensland	69	17.48	2773	13.61	
South Australia	< 5	-	626	3.07	
Tasmania	34	8.60	866	4.25	
Victoria	20	5.06	4604	22.59	
Western Australia	33	8.35	2307	11.32	
**Travel outside area for visit** ^**4**^					
Yes	33	8.35	969	4.8	0.001
**Number of subfertility/infertility encounters**					
1	285	72.15	14405	70.7	0.672
2	67	16.96	3817	18.7	
>=3	43	10.88	2160	10.6	

SEIFA, Socio-Economic Indexes for Areas

^a^Note: Percentages for characteristics may not sum to 100 due to rounding

^b^Geographical remoteness category of practice location. Based on Australian Bureau of Statistics (ABS) Australian Statistical Geography Standard (ASGS) remoteness areas – assigned according to practice postcode

^c^Geographical remoteness category of patient residence. Based on ABS ASGS remoteness areas – assigned according to postcode of patient’s residence

^d^Geographical remoteness category of practice location differs from geographical remoteness category of patient residence.

**Table 2:** Prevalence of subfertility/infertility encounters per 1,000 females, stratified by clinical and demographic characteristics, and by Aboriginal and Torres Strait Islander status

Characteristics	Encounters with Aboriginal and Torres Strait Islander females		Encounters with Non-Indigenous females		Rate Ratio^1^
	Per 1,000	95% CI	Per 1,000	95% CI	
**All patients**	12.37	11.18, 13.65	16.62	16.39, 16.85	0.74
**Age group (years) at first subfertility/infertility encounter**					
18–24	11.82	9.67, 14.31	5.48	5.19, 5.79	2.16
25–29	20.47	16.90, 24.57	18.53	17.95, 19.12	1.10
30–34	19.06	15.28, 23.48	30.90	30.14, 31.67	0.62
35–39	12.82	9.48, 16.95	30.41	29.60, 31.24	0.42
40–44	8.30	5.56, 11.93	17.58	16.93, 18.26	0.47
45–49	1.63	0.75, 3.10	3.54	3.31, 3.78	0.46
**Rurality** ^**2**^					
Major city	11.70	9.87, 13.77	17.43	17.15, 17.72	0.67
Inner/Outer Regional	13.26	11.63, 15.05	14.96	14.56, 15.36	0.89
Remote/Very Remote	7.69	3.69, 14.14	14.46	12.89, 16.18	0.53
Missing	4.57	0.16, 25.44	13.09	10.74, 15.80	0.35
**Relative socio-economic advantage and disadvantage (SEIFA quintiles)**					
First quintile (most disadvantaged)	12.52	10.28, 15.10	15.24	14.62, 15.86	0.82
Second quintile	14.38	12.03, 17.07	16.62	16.06, 17.20	0.87
Third quintile	11.46	9.17, 14.15	16.74	16.25, 17.24	0.68
Forth quintile	11.16	8.20, 14.84	16.78	16.30, 17.27	0.67
Fifth quintile (most advantaged)	9.49	5.80, 14.66	17.24	16.79, 17.71	0.55
**Commonwealth concession card**					
Yes	11.58	10.04, 13.29	10.91	10.50, 11.34	1.06
No	15.24	12.94, 17.83	19.36	19.04, 19.68	0.79
Not recorded	8.61	6.03, 11.92	14.00	13.54, 14.48	0.62
**Smoking status**					
Never smoked	16.34	13.46, 19.64	21.40	20.97, 21.85	0.76
Ex-smoker	19.26	15.32, 23.91	25.98	25.24, 26.74	0.74
Smoker	10.02	8.61,11.59	10.54	10.23, 10.84	0.95
Not recorded	7.02	4.22, 10.96	10.71	10.22, 11.21	0.66
**Comorbid conditions**					
Asthma	15.64	12.79, 18.93	19.06	18.38, 19.76	0.82
Anxiety	19.54	16.51, 22.97	20.84	20.23, 21.46	0.94
Depression	15.26	12.99, 1782	18.76	18.23, 19.30	0.81
Hypertension	11.16	7.36, 16.24	16.49	15.46, 17.56	0.68
Hyperlipidaemia	12.20	5.58, 23.15	19.21	17.22, 21.37	0.63
Hypercholesterolaemia	9.75	4.87, 17.45	16.93	15.53, 18.42	0.58
Diabetes	10.73	6.46, 16.75	16.28	14.63, 18.08	0.66
Polycystic ovary syndrome	64.80	51.42, 80.48	66.31	63.82, 68.88	0.98

SEIFA, Socio-Economic indexes for areas

^1^Rate ratio is the Aboriginal and Torres Strait Islander rate divided by the non-Indigenous rate for each strata

^2^Geographical remoteness category of patient residence. Based on ABS ASGS remoteness areas – assigned according to postcode of patient’s residence

**Table 3:** Management of subfertility/infertility encounters

Management actions	Females with encounters for subfertility/infertility (N = 20,777)					
	Aboriginal and Torres Strait Islander (N = 395)			Non-Indigenous (N = 20,382)		
	N	%		N	%	P-value
**Risk factor check**						
Blood pressure	109	27.59		5471	26.84	0.738
Body mass index	154	38.98		5175	25.39	< 0.001
Blood sugar levels	18	4.56		498	2.44	0.008
**Medication**						
Ovulation induction agents^1^	13	3.29		435	2.13	0.101
Metformin	13	3.29		497	2.44	0.278
**Clinical investigation**						
Pelvic ultrasound	96	24.30		4,056	19.90	0.030
Hysterosalpingogram	< 5	< 1.0		154	< 1.0	0.428
Hysteroscopy	0	0.0		53	< 1.0	0.361
Thyroid Function	136	34.43		7,201	35.33	0.711
Haemogloblin	140	35.44		6,672	32.73	0.256
Progesterone	125	31.64		5,903	28.96	0.244
Follicle Stimulating Hormone	121	30.63		5,325	26.1	0.044
Leutinising Hormone	126	31.89		5,228	25.65	0.005
Oestradiol	65	16.45		3,032	14.88	0.383
Iron studies	79	20.00		4,202	20.62	0.764
Prolactin	74	18.73		3,349	16.43	0.222
Testosterone	59	14.93		2,030	9.96	0.001
Sex hormone binding globulin	44	11.13		1,477	7.25	0.001
Free androgen index	35	8.86		1,386	6.80	0.003
MMR Serology	45	11.39		3,155	15.48	0.026
Glucose	64	16.20		2,757	13.53	0.124
HbA1c	25	6.32		695	3.41	0.002
Anti-Mullerian Hormone	11	2.78		1,435	7.04	0.001
Vitamin D	30	7.59		2,354	11.55	0.015

MMR, Measles, Mumps, and Rubella; HbA1c, hemoglobin A1C

Note: Percentages for management actions may not sum to 100 due to rounding. MMR, measles mumps and rubeola; HbA1c, A hemoglobin A1c

^1^Ovulation induction agents include clomifene and letrozole

## Discussion

In this large, nationally representative sample of reproductive-aged females, 1.5% of female patients with two or more GP encounters during the study period presented with a concern of subfertility/infertility. Despite Aboriginal and Torres Strait Islander females having higher rates infertility-related risk factors, including obesity, STIs and PCOS [10], were less likely to present to general practice with a concern of subfertility/infertility than their non-Indigenous counterparts. While no differences were observed in the frequency of prescribing of specific infertility medications between Aboriginal and Torres Strait Islander females and non-Indigenous females, Aboriginal and Torres Strait Islander females were more likely to receive a pelvic ultrasound as well as tests for LH, testosterone and HbA1c, yet less likely to receive an MMR and AMH test. To the authors knowledge, this is the first study aimed at investigating the management of subfertility and infertility among Aboriginal and Torres Strait Islander females in the general practice setting.

Aboriginal and Torres Strait Islander females with concerns of subfertility/infertility at general practice encounters were more likely to live in Inner/Outer regional areas and more socioeconomically disadvantaged regions. They were also more likely to hold a Commonwealth concession card. This is consistent with national data showing a relatively higher proportion of Aboriginal and Torres Strait peoples living outside major cities and high rates of social disadvantage when compared with other Australians. Aboriginal and Torres Strait Islander females were younger, with 55.7% of females under the age of 34 years, compared with 26.3% for non-Indigenous females. The higher rate of encounters among younger Aboriginal and Torres Strait Islander females may reflect the tendency to commence childbearing at younger ages (average age of Aboriginal and Torres Strait Islander females at first birth is 26 years compared to 31 years for non-Indigenous Australians [14]), and thus proportionally more females in these younger age groups actively attempting a pregnancy than in the general population. Alternatively, the higher rates may indicate young Aboriginal and Torres Strait Islander females are disproportionately affected by infertility, and therefore have a greater need for seeking treatment.

The lower rates of subfertility/infertility encounters for Aboriginal and Torres Strait Islander females seen in this study is consistent with the results of the BEACH program by Chambers and colleagues, which reported a consultation rate of 3.9 per 1,000 Aboriginal and Torres Strait Islander females, compared with 5.9 per 1,000 non-Indigenous females [9]. Interestingly, rates for each population group in the Chambers et al., study were lower than this study, which may reflect differences in the classifications of subfertility/infertility, population characteristics, and sampling methodologies used by each study (e.g., randomly sampled GPs for BEACH program vs. non-random sampling of practices to MedicineInsight). Estimates for non-Indigenous Australians observed in the study by Chambers et al. are more similar to those reported in other countries. A UK study using a primary care database containing information for approximately 1.7 million women of reproductive age, identified 3.3 per 1,000 women as having a clinically recorded fertility problem [15]. However, comparisons between general practice activity in Australia and the UK should be done with caution as the primary health systems in each country differ in their capacity, funding, and logistics [16].

Whilst speculative, it is also possible that the lower prevalence of subfertility/infertility encounters among Aboriginal and Torres Strait Islander females reflect preferences for different models of care. Although MedicineInsight includes data from Aboriginal-Community Health Organisations (ACCHOs) and Aboriginal Medical Services (AMSs), MedicineInsight does not identify these clinics, hence the number of ACCHOs/AMSs participating in MedicineInsight is unknown. If there are in fact only a handful of participating ACCHOs/AMSs, then it is possible that the lower fertility consultations among Aboriginal and Torres Strait Islander females reflect a preference to attend ACCHOs/AMSs over mainstream general practices, when raising infertility-related concerns with their GP. Indeed, when Aboriginal-specific primary health care services exist, the community prefers to and does use them, even if it means travelling considerable distances to access their ACCHOs/AMSs and bypassing several mainstream services en route [17, 18]. Accordingly, future research efforts in this area should include plans to cooperate with, or otherwise obtain de-identified information from ACCHOs and possibly other clinical services which provide primary health care to Aboriginal and Torres Strait Islander peoples (e.g., Royal Flying Doctor Service) about women’s presentations to those clinics with subfertility/infertility concerns. This will provide a more complete picture of infertility among Aboriginal and Torres Strait Islander peoples and insight into their preferences for Indigenous-specific or mainstream services for infertility, subfertility and, related health issues.

Rates were highest among Aboriginal and Torres Strait Islander females living in regional areas (13.26 per 1,000), followed by Major cities (11.70 per 1,000) and Remote/Very remote areas (7.69 per 1,000). There was less variation for non-Indigenous females, but the lowest rates were still found in Remote/very areas. This is unsurprising as it is well established that Australians living in rural and remote areas have poorer access to and use of health services compared with people living in metropolitan areas [19]. People living in rural and remote areas are more likely to face barriers to accessing a GP, including GP unavailability, longer wait times due to lower GP-to-patient ratios, and patient barriers such as travel, time and cost [19]. Other types of specialists are considerably less accessible in remote areas, including fertility specialists, and the few registered assisted reproductive clinics are located predominately in major or regional cities [20]. This means Australians who reside in remote areas are often required to travel long distances, making the frequent appointments for ART difficult. Anticipation of these logistical challenges and perception that they are too great to be overcome may deter people from initiating fertility discussions with their GP. Given that in this study Aboriginal and Torres Strait Islander females living in remote areas were about half as likely as their non-Indigenous counterparts to have a record of infertility-related concerns, we suggest that remoteness has a stronger impact on access to ART for the Aboriginal and Torres Strait Islander population than for the non-Indigenous population.

In addition to the logistic, geographic and economic barriers for patients, it is likely that cultural and social factors also influence decisions and choices about fertility treatment, and whether it is medicalised or responded to in other culturally appropriate ways. To date, little research has focused on cultural understandings of infertility, making it an important area for future research.

Aboriginal and Torres Strait Islander females from more socioeconomically disadvantaged areas had higher rates of subfertility/infertility encounters than their counterparts from more advantaged areas. An opposite trend was observed for non-Indigenous females, witencounters higher in the most advanataged areas than in the more disadvantaged areas. Generally, Australians in lower socioeconomic groups are at greatest risk of poor health and visit GPs more often than other Australians [21]. However, Aboriginal and Torres Strait Islander peoples tend to experience worse access to general practice relative to need than non-Indigenous people [22]. The low number of GP infertility consultations among Aboriginal and Torres Strait Islander females from more advantaged areas could represent a true finding of lesser need, but may reflect barriers to access for this group beyond socio-economic status (e.g., language barriers, experiences of discrimination and racism, cultural differences in constructs of health) or attendance at ACCHOs [23].

Aboriginal and Torres Strait Islander females presenting with concerns about infertility or subfertility were less likely to have a record of infertility-related comorbid conditions, most notably hypertension, hyperlipidemia and hypercholesterolaemia, than non-Indigenous females. This is inconsistent with national data which demonstrates that the rates of chronic conditions are markedly higher among Aboriginal and Torres Strait Islander peoples than among non-Indigenous people [24]. In contrast, rates of polycystic ovary syndrome (PCOS) were almost double in Aboriginal and Torres Strait Islander females in this study, which is consistent with previous research demonstrating a higher rate and greater severity in this population [25, 26]. PCOS is associated with an increased risk of infertility, as well a woman’s likelihood of developing insulin resistance and other cardiovascular disease (CVD) risk factors, particularly if not recognised and managed properly. This reinforces the importance of screening for PCOS and a range of comorbid conditions in women with subfertility/infertility, including psychological disorders, as part of routine care. Failure to do so may exacerbate infertility, lead to more complex treatments, and lengthen the overall journey to conception.

For individuals above a healthy weight, lifestyle interventions promoting weight loss are recommended as first line therapy [27, 28]. Over a third (39%) of all Aboriginal and Torres Strait Islander females presenting with infertility or subfertility had their BMI recorded within the 6 months prior to the encounter of interest, compared to a quarter (25.4%) of non-Indigenous females, suggesting room for improved weight screening in both populations. Beyond lifestyle interventions, treatment falls into three main categories: (1) medical treatment to restore fertility (e.g., the use of medications to induce ovulation); (2) surgical treatment to restore fertility (e.g., laparoscopy for ablation of endometriosis); and (3) assisted reproductive technology (ART) [27, 28]. Regarding medications, the most frequently prescribed medication in this study, regardless of Indigenous status, was metformin - a widely accepted first line treatment of T2D. This may reflect the presence of comorbid diabetes among females, rather than metformin itself being used for the management of infertility, although there is evidence that metformin improves ovulation in women with PCOS [29]. Indeed, the 2018 international guidelines for the assessment and management of PCOS recommends metformin for women with PCOS and raised BMI, impaired glucose tolerance and/or adolescents [30] and for the management of anovulation in women with PCOS with anovulatory infertility and no other infertility factors. As metformin is cheap and accessible and is associated with a reduced risk of multiple pregnancies and certain pregnancy-related complications [31], it may be used for infertility more frequently in regional and remote areas. However analyses of the MedicineInsight dataset by remoteness is required to confirm or refute this. Unsurprisingly, clomifene was the second most prescribed medication (1.87%) for both populations. Globally, clomifene is the most common medication used for ovulation induction, since it is inexpensive, highly effective and user-friendly [27, 28]. However, clomifene prescriptions were sparsely recorded, most likely reflecting restrictions on GP’s being able to prescribe this medication in place in several jurisdictions.

Pelvic ultrasound was the most common type of imaging for both populations, however, was more likely to be for Aboriginal and Torres Strait Islander females (24.3% vs. 19.9%). Records of hysterosalpingograms and hysterosalpingo-contrast sonography were much less common (0.5% vs. 0.8% and 0% vs. 0.3%, respectively). In comparison, only 9.3% of patients in study by Chambers et al., had a record of a specified image test (one of pelvis ultrasound, hysterosalpingogram, hysterosalpingo-contrast sonography or transvaginal ultrasound)[9].

This apparent increasing use of diagnostic medical imaging in infertility management is in line with the significant increase in diagnostic imaging in Australia general practice settings but could also reflect increased awareness of the need to investigate for PCOS.

Aboriginal and Torres Strait Islander females were more likely to receive tests for LH (31.9% vs. 25.7%), testosterone (14.9% vs. 10.0%), HbA1c (19.2% vs. 15.2%); and FAI (8.9% vs. 6.8%); but less likely receive an MMR serology (11.4% vs. 15.5%) and AMH test (2.8% vs. 7%), than non-Indigenous females. Given AMH testing is not covered by Medicare, typically costing around $80, the lower AMH testing rate among Aboriginal and Torres Strait Islander peoples may reflect the relative socioeconomic disadvantage experienced by this group or the tendency of women to give birth at younger ages.

### Strengths and limitations

The main strength of this study is the size and national coverage of the MedicineInsight dataset, and that the cohort is broadly representative of the Australian patient population in terms of age, sex, socioeconomic-status and with respect to Indigenous status (3.0% vs. 3.3%) [11]. This study includes data from the largest and most representative sample of reproductive-aged females attending Australian general practice clinics, including around 32,000 Aboriginal and Torres Strait Islander females. The use of clinical records reduces the subjective bias found in self-reported health surveys, since clinical records comprise GP-identified diagnoses, objective laboratory and medicines prescribed to patients.

Despite the strengths, this study is subject to several limitations. The first and major limitation of this study is that the extent of participation of ACCHOs and AMSs in MedicineInsight is unknown. Given that these services are often the preferred model of health care for Aboriginal and Torres Strait Islander peoples [18], it possible that the frequency of presentation for fertility concern may be higher in the ACCHO sector. This warrants investigation in further studies. Nevertheless, the dataset still has high representation of Aboriginal and Torres Strait Islander females. Second, it is important to acknowledge the inherent limitations in routinely collected data as described elsewhere [32]. Key issues to be considered relate to the accuracy, completeness and precision of data. However, to maximise the data quality, contributing data to MedicineInsight are subject to specific MedicineInsight data quality requirements, which includes extensive data cleaning procedures [11]. To further improve data, the included cohort was restricted to individuals who visited the same general practice more than once during the study period. Third, for privacy reasons, MedicineInsight does not include data from the unstructured ‘progress notes’ section, which may contain further relevant information related to infertility/subfertility encounters such as referral to other health care professionals. Fourth, MedicineInsight is currently unable to link patients across different practices, and consequently, patients who attended multiple MedicineInsight practices may be recorded more than once. However, it has been estimated that the rate of duplication in the dataset is less than 4% of patients [11]. Fifth, whilst the search terms and synonyms used for subfertility/infertility in this study were comprehensive and captured most patients presenting with related concerns, there is likely to be variation in how GPs record infertility-related consultations which means some may have been missed or recorded incorrectly. However extensive coding was undertaken to minimise this risk. Sixth, MedicineInsight does not currently include referrals provided by GPs to secondary care. However, in the study by Chambers et al., referrals to secondary care occurred in approximately 40% of female consultations [9], and a UK study reported three quarters of couples, presenting for the first time to their GP with a fertility problem, were referred for specialist help [33] [18]. Finally, the cross-sectional nature of this study means that the longitudinal pathway of each patient and temporal relationships has not been explored. A longitudinal analysis of this data is planned.

## Conclusion

This study provides important insights into the management of subfertility/infertility among Aboriginal and Torres Strait Islander females attending general practice. The lower rate of encounters for Aboriginal and Torres Strait Islander females may reflect poorer access to appropriate primary care services for this group but could also reflect other culturally appropriate responses to infertility within communities. Additionally, the high encounter rates among younger Aboriginal and Torres Strait Islander females may reflect early childbearing, however it could also indicate that these females are at higher risk of infertility and thus more likely to seek treatment. Findings could be useful for the development of targeted education strategies to improve awareness of infertility among community members and clinicians. Findings could also be useful in planning effective interventions to support GPs in the equitable and optimal management of infertility/subfertility in Australia e.g., development of infertility clinical practice guidelines. Finally, to address the evidence gaps regarding management of infertility in the Aboriginal-specific primary health care sector, future efforts should be made to cooperate with or otherwise obtain de-identified information from these organisations, and possibly other clinical services (e.g., Royal Flying Doctor Service).

## Data Availability

The datasets supporting the conclusions of this article are included within the article. The data extraction algorithms used in this study are available from the corresponding author (Emily Gilbert, Email: emily.gilbert@cdu.edu.au) by request.

## Footnotes

1 MedicineInsight uses the terms sex and gender interchangeably and presents sex/gender information as a single outcome. In line with the language used by MedicineInsight, this paper has used the term “female” to describe the cohort, however it should be noted that the authors acknowledge that sex and gender are distinct concepts, and that sex and gender do not automatically align. Further, due to the small numbers of intersex or indeterminate patients, these patients were included the baseline population, but not in further analyses. Only male and female records are included in the database.
